# Editorial: Applications of artificial intelligence in forensic mental health: opportunities and challenges

**DOI:** 10.3389/fpsyt.2024.1435219

**Published:** 2024-06-12

**Authors:** Giovanna Parmigiani, Gerben Meynen, Toni Mancini, Stefano Ferracuti

**Affiliations:** ^1^ Department of Human Neurosciences, Faculty of Medicine and Dentistry, Sapienza University of Rome, Rome, Italy; ^2^ Willem Pompe Institute for Criminal Law and Criminology, School of Law, Faculty of Law, Economics, and Governance, Utrecht University, Utrecht, Netherlands; ^3^ Faculty of Humanities, Vrije Universiteit (VU) Amsterdam, Amsterdam, Netherlands; ^4^ Department of Computer Science, Faculty of Information Engineering, Computer Science and Statistics, Sapienza University of Rome, Rome, Italy

**Keywords:** artificial intelligence, forensic psychiatry, machine learning (ML), offenders, ethical challenges

In recent years, growing attention has been paid to the possible application of artificial intelligence (AI) techniques and machine learning (ML) in clinical and forensic settings.

AI methods based on Knowledge Representation and Automated Reasoning (KR&R), Model Checking (MC) as well as Machine (deep-)Learning (ML) have been employed for the development of predictive quantitative models for, e.g., biochemical reactions, human pathophysiology, and many other domains.

In the forensic field, discriminative AI has been applied for predicting risk of aggression (Kirchebner et al., 2020; Gou et al., 2021; Parmigiani et al., 2022; Watts et al., 2021), crime recidivism (Tollenaar and van der Heijden, 2019), and forecasting future offences (Watts et al., 2021). In addition, AI has been used to inform decisions about sentencing, parole, probation or pretrial risk assessment, raising several legal and ethical concerns regarding, among others, fairness, accountability and transparency (Tortora et al., 2020). These concerns stem, for instance, from the finding that some algorithms contained race and gender bias (Barabas et al., 2018), the fact that the scores may be misapplied and misinterpreted by judges and practitioners (Hannah-Moffat, 2015), and proprietary issues which can contribute to a lack of transparency (Barabas et al., 2018).

This Research Topic aims to present cutting-edge studies on the application of AI techniques in the forensic mental health field, including research on ethical challenges such as those related to the need to ensure non-discrimination, the “fair process” and the values of transparency and comprehensibility of decision-making processes.

The contributing works are two original articles (Bender et al. Machetanz et al.), one perspective article (Starke et al.) and one conceptual analysis article (Tortora).

Bender et al. employed supervised machine learning algorithms in order to identify distinguishing factors between 269 offenders and 184 non-offenders affected by both schizophrenia spectrum disorders and substance use disorder. The ML model, with an AUC of 0.88, found the most influential distinguishing factors to be: failures during opening (defined as rule violations during a permitted temporally leave), nonadherence to treatment, social isolation, and the absence of antipsychotic prescription and no outpatient psychiatric treatments before the current hospitalization.

Similarly, Machetanz et al. used discriminative AI (through the use of supervised ML algorithms), to build a model, with an AUC of 0.86, able to investigate the clinical key factors discriminating 370 offenders and 370 non-offenders diagnosed with a schizophrenia spectrum disorder. The most influential predictors were: olanzapine equivalent at discharge, history of antipsychotic prescription, a history of antidepressant, benzodiazepine or mood stabilizer prescription, medication compliance, outpatient treatment(s) in the past, and the necessity of compulsory measures.

Starke et al. wrote a perspective article focused on the ethical challenges of using AI methods in forensic psychiatry. The authors argue that, given the importance and influence of environment and social circumstances on the onset and course of psychiatric disorders, we should not overlook these factors when developing predictive algorithms (from the collection of data in the training set, to the selection of ML methods, and the formulation of the explainability requirements).

Finally, Tortora in her conceptual analysis article, examined the potential of Generative AI in reshaping forensic mental health practice in comparison with the Discriminative AI paradigm. Next, she addresses ethical and legal issues associated with Generative AI application, such as, biased and stereotyped outputs, lack of transparency, ‘hallucinations’ and facts fabrication, highlighting the risks of the spread of misinformation and the reinforcement of discriminatory and criminalizing narratives and stereotypes.

## Conclusions

This Research Topic provides a collection of studies on the application of AI techniques in the forensic mental health setting. While underlining the potential of both discriminative and generative AI for this domain, it also emphasizes associated risks and ethical concerns. We hope this Research Topic will be a helpful reference for forensic practitioners who must deal with the increasing role of IA in their work, and that it will provide a stimulus for much-needed further research on this fascinating theme.

## Author contributions

GP: Writing – original draft, Writing – review & editing. GM: Writing – review & editing. TM: Writing – review & editing. SF: Writing – review & editing.
